# Multicontrast MRI Quantification of Focal Inflammation and Degeneration in Multiple Sclerosis

**DOI:** 10.1155/2015/569123

**Published:** 2015-07-29

**Authors:** Guillaume Bonnier, Alexis Roche, David Romascano, Samanta Simioni, Djalel Eddine Meskaldji, David Rotzinger, Ying-Chia Lin, Gloria Menegaz, Myriam Schluep, Renaud Du Pasquier, Tilman Johannes Sumpf, Jens Frahm, Jean-Philippe Thiran, Gunnar Krueger, Cristina Granziera

**Affiliations:** ^1^Advanced Clinical Imaging Technology Group, Siemens, Innovation Park, EPFL, 1015 Lausanne, Switzerland; ^2^LTS5, École Polytechnique Fédérale de Lausanne, 1015 Lausanne, Switzerland; ^3^Department of Neurology, Centre Hospitalier Universitaire Vaudois and University of Lausanne, 1011 Lausanne, Switzerland; ^4^Department of Radiology, Centre Hospitalier Universitaire Vaudois and University of Lausanne, 1011 Lausanne, Switzerland; ^5^Department of Radiology and Medical Informatics, University of Geneva, 1211 Geneva, Switzerland; ^6^Medical Image Processing Laboratory (MIPLAB), Institute of Bioengineering, EPFL, 1015 Lausanne, Switzerland; ^7^Department of Computer Science, University of Verona, 37134 Verona, Italy; ^8^Biomedizinische NMR Forschungs GmbH, Max Planck Institute for Biophysical Chemistry, 37077 Goettingen, Germany; ^9^Healthcare Sector IM&WS S, Siemens Schweiz AG, 1020 Renens, Switzerland; ^10^Laboratoire de Recherche en Neuroimagerie, Department of Clinical Neurosciences, Centre Hospitalier Universitaire Vaudois and University of Lausanne, 1011 Lausanne, Switzerland

## Abstract

*Introduction*. Local microstructural pathology in multiple sclerosis patients might influence their clinical performance. This study applied multicontrast MRI to quantify inflammation and neurodegeneration in MS lesions. We explored the impact of MRI-based lesion pathology in cognition and disability. *Methods*. 36 relapsing-remitting MS subjects and 18 healthy controls underwent neurological, cognitive, behavioural examinations and 3 T MRI including (i) fluid attenuated inversion recovery, double inversion recovery, and magnetization-prepared gradient echo for lesion count; (ii) T1, T2, and T2^*^ relaxometry and magnetisation transfer imaging for lesion tissue characterization. Lesions were classified according to the extent of inflammation/neurodegeneration. A generalized linear model assessed the contribution of lesion groups to clinical performances. *Results*. Four lesion groups were identified and characterized by (1) absence of significant alterations, (2) prevalent inflammation, (3) concomitant inflammation and microdegeneration, and (4) prevalent tissue loss. Groups 1, 3, 4 correlated with general disability (Adj-*R* ^2^ = 0.6; *P* = 0.0005), executive function (Adj-*R* ^2^ = 0.5; *P* = 0.004), verbal memory (Adj-*R* ^2^ = 0.4; *P* = 0.02), and attention (Adj-*R* ^2^ = 0.5; *P* = 0.002). *Conclusion*. Multicontrast MRI provides a new approach to infer *in vivo* histopathology of plaques. Our results support evidence that neurodegeneration is the major determinant of patients' disability and cognitive dysfunction.

## 1. Introduction

Multiple sclerosis (MS) is an inflammatory and neurodegenerative disease affecting the brain and spinal cord. The hallmark of MS is the presence of multifocal lesions or “plaques,” which are characterized by variable inflammatory, degenerative, and reparative processes [1, 2]. Plaques inflammation is widespread in the relapsing-remitting MS subtype, whereas important tissue loss is pronounced in progressive MS and in long-standing disease [3, 4]. In addition, new lesions are mostly characterized by inflammatory phenomena, leading to blood-brain barrier disruption, while older lesions show a higher proportion of neurodegeneration and/or repair processes [4, 5].

Conventional magnetic resonance imaging (cMRI) is a valuable tool to provide information about the number, location, and inflammatory “activity” of focal lesions. Nevertheless cMRI offers only limited sensitivity to focal pathology in the cortex and little insight into the nature of local damage. Nonconventional MRI techniques such as double inversion recovery (DIR, Geurts Radiology 2005) and magnetization-prepared 2 rapid gradient echo (MP2RAGE, Marques Neuroimage 2010 and Kober 2012) have proven higher sensitivity to focal cortical pathology than cMRI. Similarly, the combination of multiple cMRI contrasts improved cortical lesions detection at all field strengths (1.5 T (B. Moraal), 3 T (M. Archambault-Wallenburg), and 7 T (W. L. De Graaf)). Besides, other advanced MRI techniques have shown to be sensitive to tissue pathology in lesions, such as axonal and myelin damage (diffusion tensor imaging (DTI) and magnetisation transfer imaging (MTI)) and axonal metabolic deficits (magnetic resonance spectroscopy) [2, 6–9]. MRI relaxometry has also been extensively used to study normal-appearing brain tissue in multiple sclerosis patients (for review see [9, 10]), but only few works focused on lesions properties and heterogeneity [11, 12]. Yet, some recent postmortem studies provided strong evidence of the value of MRI relaxometry techniques to study specific aspects of plaques pathology; Bagnato et al. showed that high R2^*∗*^ values in the periphery of white matter (WM) lesions correlated with iron accumulation in macrophages/microglia whereas high R2^*∗*^ inside the WM plaque had the appearance of iron aggregates typical of microbleeds [13]. Furthermore, Tardif et al. established that myelin loss within cortical lesions was associated with a concomitant increase of T1 and T2 relaxation times and a decrease of MTI measures [14].

In this work, we combined,* in vivo* in MS patients, three relaxometry techniques (T1, T2, and T2^*∗*^), and MTI. The aims of the study were (i) to classify MS cortical and white matter lesions according to the extent of inflammatory and neurodegenerative phenomena, as measured by unconventional MRI and (ii) to assess the clinical impact of MRI measures of lesion pathology in a cohort of relapsing-remitting multiple sclerosis patients.

## 2. Methods

### 2.1. Study Population

Thirty-six patients with relapsing-remitting MS (RRMS) and eighteen age-matched healthy controls (HC) were enrolled in this cross-sectional study between January and December 2012. The age of the patients was 34.8 ± 9.2 years (mean ± standard deviation (SD)) and gender ratio was 24/12, women/men. HC aged 33 ± 9.7 years and had a gender ratio of 9/9. The time elapsed since the first symptoms was 33.3 ± 21 months (range: 2–70 months) and the time since disease diagnosis was 27.1 ± 18 months (range 0–59 months). Immunomodulatory treatment, consisting in high dose interferon-beta (IFN-*β*) or fingolimod, was administered to thirty patients out of thirty-six patients (83%) for at least 3 months. No patient had received corticosteroid therapy within the three months preceding the study. The study was approved by the ethics committee of the Lausanne University Hospital (CHUV). Written, informed consent was obtained from each subject.

### 2.2. Clinical Assessment

Verbal and spatial memory, sustained attention, information processing speed, and verbal fluency on semantic cues were assessed at the time of MRI for each subject using the Brief Repeatable Battery of Neuropsychological Tests (BRB-N) [15]. Depression and fatigue were quantified using the Hospital Anxiety and Depression Scale (HAD) [16] and the Fatigue Scale for Motor and Cognitive Functions (FSMC) [17]. Finally, the Expanded Disability Status Scale (EDSS [18]) and the Multiple Sclerosis Functional Composite (MSFC [19]) scores were evaluated to quantify disability and motor performances.

### 2.3. MRI Acquisition

All subjects underwent MRI examinations on a 3 T Siemens Trio (Siemens, Erlangen, Germany) equipped with a 32-channel head coil. MRI protocol details were previously reported in [20] and summarized in Table  1s (supplementary data) (see Supplementary Material available online at http://dx.doi.org/10.1155/2015/569123). In summary, a 3D magnetization-prepared acquisition with gradient echo (MPRAGE) was acquired for automatic brain tissue and atlas-based segmentation [21–23]; 3D fluid attenuated inversion recovery (3D FLAIR), 3D double inversion recovery (3D DIR), and 3D MP2RAGE [11] were acquired for lesion detection and segmentation. The MP2RAGE sequence additionally provided whole-brain T1 relaxometry [24]. T2^*∗*^ relaxometry maps were obtained using 32 echoes and a correction method based on an estimated B0 field map [25]. Magnetization transfer ratio (MTR) maps were derived from the T2^*∗*^ data, after registration of echoes with (MT) and without MT pulse (M0) (MT pulse flip angle: 220°; duration: 4000 ms; pulse offset: 2000 Hz; and spoiler moment: 25000 us*∗*mT/m). The magnetization transfer ratio MTR = (M0 − MT)/M0 was then computed for each echo and averaged over all echoes. For T2 relaxometry, we used a new nonlinear inverse reconstruction algorithm [26] that directly estimates a T2 and spin-density map from a train of undersampled spin echoes. The acquisition of T2 relaxometry maps was performed with a spatial resolution, which is lower than the one achieved for the other MRI contrasts and maps. Nevertheless, the current protocol appears to have quite similar resolution compared to recently published T2 mapping sequences [27] and was optimized to achieve the best T2 maps quality in clinically compatible scanning times.

**Figure 1:**
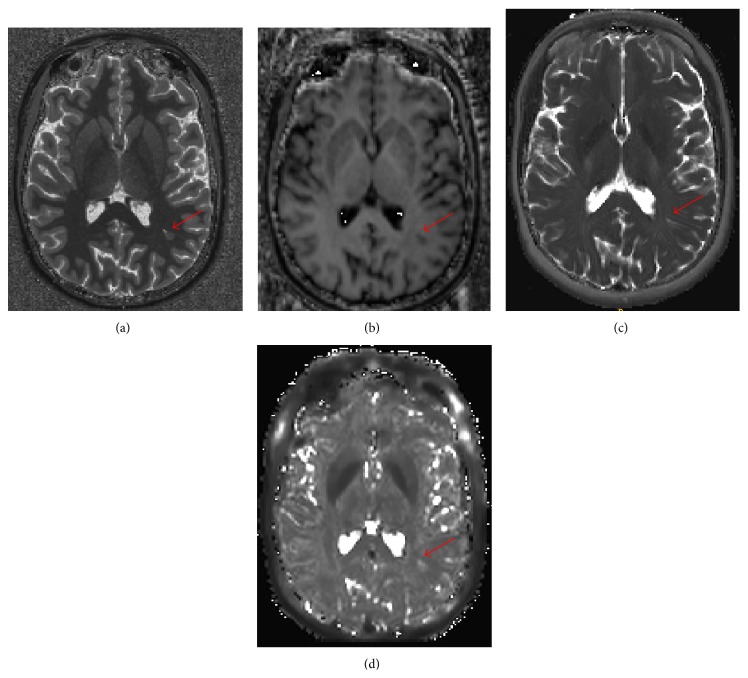
T1 map (a), MTR (b), T2 map (c), and T2^*∗*^ map (d) in one MS patient. An example of lesion is shown by a red arrow.

Visual inspection of image quality was performed in all cases. An example of T1, T2, and T2^*∗*^ and MTR maps is reported in Figure 1.

**Figure 2:**
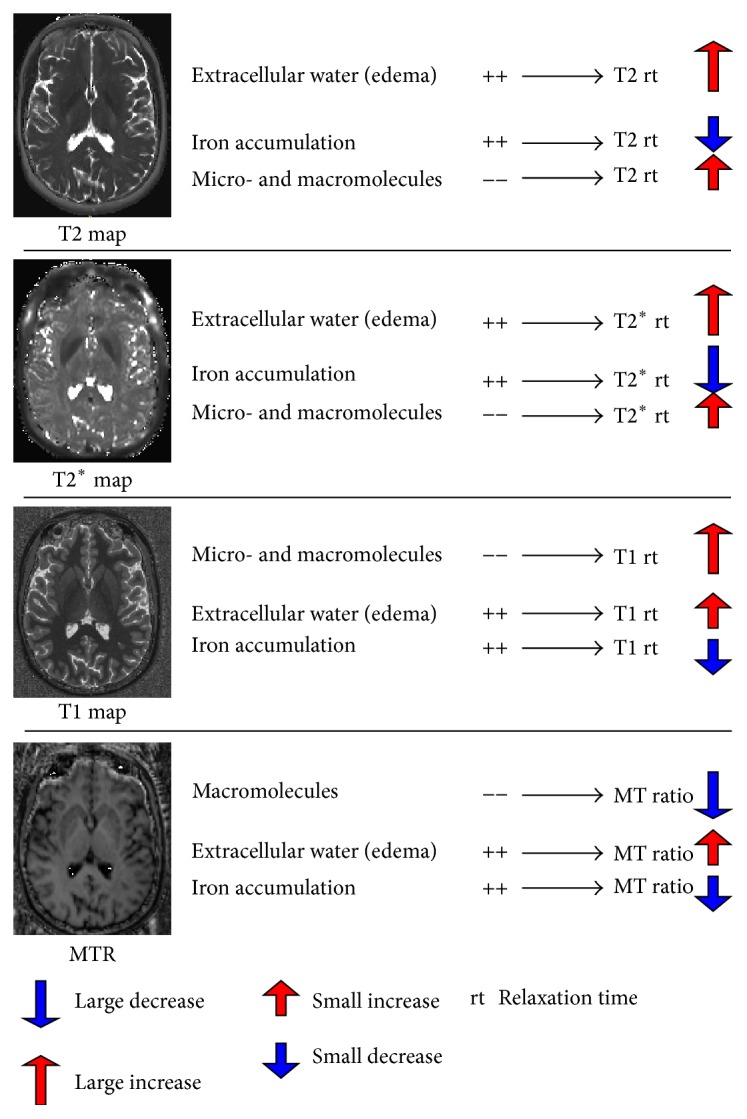
Biological interpretation of quantitative and semiquantitative MRI contrasts.

The biological interpretation of changes in T1, T2, and T2^*∗*^ relaxation times (rt) and MTR was summarized in Figure 2 and previously reported in detail [20].

### 2.4. Image Analysis

Rigid registrations with BSpline interpolation were performed, using Elastix C++ [28], to register (i) the T2 maps to the T1 maps (MP2RAGE) and (ii) the T2^*∗*^ maps, MPRAGE, FLAIR, and DIR images to one of the inverted contrasts of the MP2RAGE sequence.

Cortical and WM MS lesions were manually identified in patients by an experienced neurologist (CG) and a radiologist (DR) using 3D FLAIR, 3D DIR, and MP2RAGE images, as previously reported [20, 22, 24]. Manual contours were generated for each lesion by a trained technician for each contrast. As reported by [11, 20], we merged the lesions extracted from FLAIR, DIR, and MP2RAGE to obtain a final union lesion mask for each subject. Lesion volumes were computed and normalized by total intracranial volume as obtained using an in-house software [20, 29]. Only lesions with more than 10 voxels size were included in the analysis. Lesion masks were then registered to MP2RAGE space using the registration parameters described above and mean T1, T2^*∗*^ and MTR were calculated for each lesion.

In order to assess the mean distribution of T1, T2, and T2^*∗*^ rt and MTR in HC brain tissue, we segmented lobar WM and cortical GM (frontal, parietal, occipital, and temporal) as well as cerebellar WM/GM from the MPRAGE images using an in-house software based on variational expectation-maximization tissue classification [20, 29].

To compare lesion MRI properties in patients with the corresponding tissue in HC, we calculated a *z*-score for each contrast in each lesion (e.g., for T1 data):

(1) $$\begin{array}{c} z_{T1}=\frac{1}{N}\sum_{v\in l}\frac{I_{T1}\left(v\right)-\mu_{T1}\left(L_{l},T_{l}\right)}{\sigma_{T1}\left(L_{l},T_{l}\right)}, \end{array}$$

where *z*_T1_ corresponds to the T1 lesion *z*-score (*z*), *l* to the lesion voxels, *N* to a normalisation term, *I*_T1_ to the T1 map, and *μ*_T1_(*L*_*l*_, *T*_*l*_) and *σ*_T1_(*L*_*l*_, *T*_*l*_) to the mean and the standard deviation of the T1 map in the lobe *L*_*l*_ and tissue *T*_*l*_ (i.e., WM or GM) in the HC group, corresponding to the lesion location and type.

Considering the continuous distribution (without distinct cluster) of lesions *z*-scores in each contrast, we classified the lesions into 3 groups as follows: (i) *z* very low (*z* < −2), (ii) *z* very high (*z* > 2), and (iii) *z* close to the HC distribution (−2 ≤ *z* ≤ 2). The thresholds were chosen considering the fact that more than 95 percent of the *z*-scores belong to the interval [−2,2] in a normal distribution and that values beyond this interval reflect significant differences in patients compared to controls (*P* < 0.05).

**Figure 3:**
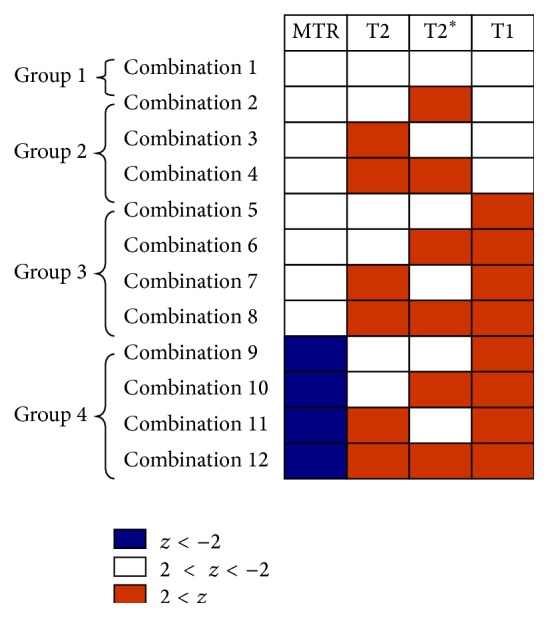
Groups and contrasts combinations of MS lesion *z*-scores for T1, T2, and T2^*∗*^ and MTR contrasts, as observed in our cohort of RRMS patients. Blue: parameter decrease; red: parameter increase. Group 1: lesions with no significant q/sq MRI contrasts changes; Group 2: lesions with prevalent inflammatory oedema; Group 3: lesions with prevalent tissue degeneration with or without inflammation; and Group 4: lesions with prevalent tissue loss.

Last, for each subject, all existing combinations (Figure 3) of *z* were computed for all contrasts (e.g., combination 1 = *z*_T1_ > 2, *z*_T2_ > 2, *z*_T2^*∗*^_ > 2, and *z*_MTR_ < −2; combination 2 = *z*_T1_ > 2, *z*_T2_ > 2, −2 < *z*_T2^*∗*^_ < 2, and *z*_MTR_ < −2, etc.) and mean lesion volume (MLV) was assessed for each combination (total normalized lesion volume/number of lesions).

### 2.5. Statistical Analysis

#### 2.5.1. Between-Groups Comparisons of Subjects' Demographics and Clinical Scores

Differences in age, gender, education, and clinical performance were assessed using a nonparametric ANOVA (Kruskal-Wallis test) among HC and MS patients.

#### 2.5.2. Multivariate Linear Regression of Clinical Scores in Patients with T1, T2, T2^**∗**^ and MTR in Lesions

A multivariate linear regression of clinical scores was performed using a general linear model (GLM) applied to MLV in each combination of contrasts. Age, gender, educational years, anxiety, and depression scores (HAD) were considered as covariates, since they have been reported to be linked to functional performance in MS patients [30, 31]. Cognitive scores were adapted using Box-Cox transformation to satisfy the model assumption for normality [32].

We performed eight regressions and applied a backward stepwise approach to select the best prediction model for each dependent variable (clinical scores). Bonferroni correction was applied for multiple comparisons (seven tests). “Leave-one-out” (LOO) cross-validation was applied to assess the prediction quality and robustness of each model. A* P* value < 0.05 was considered statistically significant.

All regression analyses were performed using R software (http://www.r-project.org/).

## 3. Results

### 3.1. Between-Groups Comparisons of Subjects' Demographics and Clinical Scores

No significant differences were observed between HC and MS patients in terms of age (*P* = 0.3) or gender (*P* = 0.8); however, HC had slightly higher education levels (17 ± 4 years, mean ± standard deviation) than MS patients (15 ± 3 years; *P* = 0.04).

Mean EDSS in patients was 1.6 ± 0.3 (interval: 1-2). The FSMC motor score was significantly higher in MS patients (23.1 ± 10.5) than in HC (14.8 ± 5.8; *P* < 0.02). The FSMC cognitive scores, cognitive performance, MSFC scores, and anxiety and depression scores (HAD) were not significantly different between groups (*P* > 0.1).

### 3.2. Contrasts Combinations and Lesion Combination Distribution

We found 12 *z*-scores combinations in all MS lesions (1402 lesions, Figure 3). These combinations characterised plaques with no significant contrast changes (Group 1: combination 1, 54% cortical and 46% WM lesions), prevalent inflammatory edema (Group 2: isolated increase of T2 and/or T2^*∗*^ *z*-scores, combinations 2–4, 40% of cortical and 60% of WM lesions), microdegeneration, and/or inflammatory edema (Group 3: increase in T1 and/or increase in T2/T2^*∗*^, combinations 5–8, 2% cortical and 98% WM lesions), and broad tissue loss (Group 4: strong increase in T1 and decrease in MTR *z*-scores, with or without increase in T2/T2^*∗*^, combinations 9–12, 100% WM lesions) (Figure 3).

**Figure 4:**
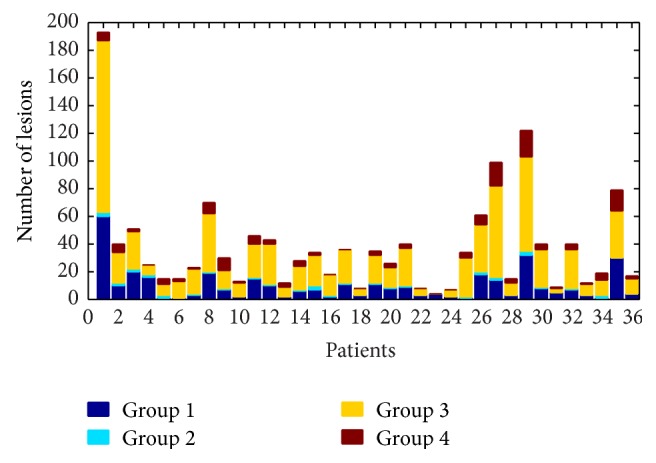
Lesion groups distribution in RRMS patients. Groups 1 and 3 account for more than 60% of all lesions and are the most represented groups in patients.

Most of the lesions (70%) showed a significantly high T1 *z*-score (Group 3 and 4) and only 27% of total number of lesions did not show any significant change in all contrasts (Group 1); 48% of lesions showed high T1 *z*-score only (Group 3), 32% exhibited high T1 *z*-score combined with high T2 or T2^*∗*^ (Group 3), and 18% were characterized by high T1 *z*-score combined with low MTR (Group 4). Group 2 containing lesions with high T2 and/or T2^*∗*^ and “nonsignificant” T1 and MTR counted less than 3% of the total number of lesions (Figure 4).

The cortical lesions represented 17% of the total number of lesions; 90% were cortical lesions Type I (mixed GM/WM) and 10% Type II (GM only) (Table 1). They mainly belong to combination 1 (85%) and combinations 2 to 8. Most of the lesions were pure white matter lesions (83%, Table 1) and appeared in all combinations.

### 3.3. Multivariate Linear Regression of Clinical Scores in Patients with T1, T2, and T2^**∗**^ and MTR in Lesions

GLM using stepwise regression revealed a highly significant association, confirmed by a cross-validation test, between lesions MRI characteristics of lesions and three clinical scores (Table 2).

(i) The MLV in combinations 8 and 9 (Group 3 and 4) together with age and depression score predicted the MSFC (general disability) score (Adj-*R* ^2^ = 0.6; *P* = 0.0005).
(ii) The MLV in combinations 5, 6, and 9 (Group 3 and 4) in conjunction with gender predicted the FV (execution) score (Adj-*R* ^2^ = 0.5; *P* = 0.002).
(iii) The MLV in combinations 1, 9, and 10 (Groups 1 and 4) predicted the SRT (verbal memory) score (Adj-*R* ^2^ = 0.4; *P* = 0.002).
(iv) MLV in combinations 1, 5, 6 and 9 (Groups 1, 3, and 4) with age and depression score predicted the SDMT (attention function) score (Adj-*R* ^2^ = 0.5; *P* = 0.004). Nevertheless, cross-validation test revealed a possible overfitting of the GLM (estimated score versus clinical score: Adj-*R* ^2^ = 0.1; *P* = 0.09).

## 4. Discussion

Current diagnostic and prognostic criteria in MS as well as clinical trials end-points are based on conventional MRI measures of lesions number, volume, and activity [33]. Nevertheless, these parameters provide only limited information about the nature and severity of tissue alterations in the central nervous system.

In fact, changes in conventional T1 and T2 signals are compatible with both inflammatory and degenerative phenomena [20]; moreover, the presence of “black holes,” considered to be a marker of permanent axonal/myelin loss [34, 35], might be also due to inflammatory extracellular edema [35] and activated microglia [36, 37]. Furthermore, gadolinium (Gd) enhancement, a conventional marker of active inflammation, does not detect active lesions with mild changes in blood-brain barrier (BBB) permeability [38] and disseminated inflammation due to activated microglia [39]. In addition, the presence of Gd uptake might reveal incomplete restoration of tight junction integrity and BBB function in inactive, noninflamed, chronic lesions [40].

We recently showed the potential of advanced MRI techniques to unravel the nature of diffuse and focal tissue pathology in MS [20]. In this work, we aimed at investigating the influence of unconventional MRI metrics of lesion pathology on patients' disability and cognition.

In accordance with previous literature at 3 T [11] we found that the majority of lesions detected in our cohort of early MS patients were located in WM (83%), a moderate number were mixed WM/GM (cortical lesion Type I) (15%), and few were purely cortical and punctiform (cortical lesion Type II) (2%) (Table 1).

We identified twelve combinations of MRI contrasts in MS lesions, which we organized into four main groups according to the predominant underlying pathology (Figure 3). Group 1 was constituted by lesions that did not show any significant contrast change, possibly due to pathophysiological causes (i.e., presence of more efficient reparative processes in early stages of disease) and/or technical aspects (lack of sensitivity/spatial resolution). The other three groups were constituted by lesions exhibiting prevalent inflammation (Group 2), microdegeneration with/without inflammation (Group 3), or predominant tissue loss (Group 4). These four groups were consistent with those reported by the histopathological “Vienna Classification” of MS lesions (Group 1: Vienna lesion type VLT 6; Group 2: VLT 2; Group 3: VLT 2/5; and Group 4: VLT 5) [41].

Interestingly, we did not observe any T1/T2/T2^*∗*^ decrease in local plaques, suggesting that no significant iron accumulation occurs in our cohort of patients. However, since we performed an average lesion analysis, this observation does not exclude the presence of local iron increase, as previously reported [13, 42].

Last, we studied the relative impact of lesion combinations/groups on clinical performance in patients. And we found that lesions with concomitant microdegeneration/inflammation or important tissue loss had a greater impact on patients' disability, executive function, and verbal memory than prevalent inflammatory lesions. This result could be due to the presence of a minority of lesions in the purely inflammatory group (Group 2), which might be due to the fact that most of the patients were benefitting of immunomodulatory/immunodepressive therapy. In addition, lesions with no significant changes in multicontrast MRI (Group 1) played an important role in verbal memory and attention. This aspect is coherent with the fact that the majority of Group 1 lesions were located in the cortical layers; yet, it could be also due to the fact that a proportion of Group 1 lesions are located in eloquent areas. In order to elucidate this last point, an ongoing study is aiming at integrating the lesion location information in the current lesion classification.

In summary, our current work provides a new approach to infer histopathological information from MS plaques and supports evidence that MRI measures of lesion pathology are strong determinants of patients' clinical performance in our cohort.

A technical limitation of this study is the low in-plane resolution of the T2 relaxation maps, compared to the other applied maps and MRI contrasts. Though we tried to overcome this limit by setting a threshold to lesion size (>10 voxels), this aspect could impact the estimations of average T2 values in small lesions. Future hardware and software improvements are required to achieve higher spatial resolution in accelerated T2 relaxometry acquisitions. Another limitation of this method is the lack of sensitivity to repair/plasticity (i.e., gliosis, axonal remodeling, etc.) as well as to other inflammatory phenomena like lymphocytic/microglia infiltration and activation. Studies focusing on the longitudinal pattern of contrasts evolution in MS lesions and the combination with other MRI contrasts (i.e., diffusion imaging) or modalities (i.e., MRI-PET) might help to overcome these limits.

## Supplementary Material

Description of the MRI acquisition protocol. TR: repetition time; TE: echo time; FoV: field of view.

## Figures and Tables

**Table 1:** Lesions count in brain hemispheres and cerebellum.

Combinations	Brain			Cerebellum		
	WM	Cortical		WM	Cortical	
		Type I	Type II		GM/WM	GM
1	161	186	7	10	11	0
2	7	8	4	0	1	0
3	10	3	0	0	1	0
4	438	7	6	24	0	0
5	3	1	0	0	0	0
6	216	0	0	1	0	0
7	50	1	1	3	0	0
8	89	0	0	1	0	0
9	51	0	0	0	0	0
10	8	0	0	0	0	0
11	34	0	0	0	0	0
12	58	0	0	1	0	0

Total no.	1125	206	18	40	13	0

%	80.24	14.69	1.28	2.85	0.93	0

**(a):** 

Predictors (*P* value)	Clinical scores						
	MSFC	FV	SRT	SDMT	Tot10/36	FSMCCog	FSMCMot
Stepwise regression							
*P* value	0.00006^*^	0.00024^*^	0.00219^#^	0.00054^*^	0.03156^†^	0.03120^†^	0.03090^†^
Corrected *P* value	0.00045^*^	0.00166^#^	0.01536^†^	0.00379^#^	0.22092^‡^	0.21840^‡^	0.21630^‡^
Adjusted-*R*	0.55050	0.45350	0.38040	0.48960	0.20990	0.24770	0.2483

Cross-validation: leave-one-out							
*P* value	0.00001^*^	0.00004^*^	0.00097^*^	0.01300^†^			
Corrected *P* value	0.00005^*^	0.00030^*^	0.00677^#^	0.09100^‡^			
Adjusted-*R*	0.43660	0.37490	0.25620	0.14360			

**(b):** 

Predictors (*P* value)	Clinical scores						
	MSFC	FV	SRT	SDMT	Tot10/36	FSMCCog	FSMCMot
*z*-scores combination							
Group 1							
Combination 1			0.0048^#^	0.0003^*^			
Group 2							
Combination 2							
Combination 3							
Combination 4							
Group 3							
Combination 5		0.0003^*^		0.0088^#^			0.0223^†^
Combination 6		0.0182^†^		0.0049^#^			
Combination 7							
Combination 8	0.0200^†^						
Group 4							
Combination 9	0.0011^#^	0.0256^†^	0.0175^†^	0.0001^*^		0.0331^†^	0.0168^†^
Combination 10			0.0057^#^				
Combination 11							
Combination 12							

Covariates							
Age				0.0056^#^			0.0144^†^
Gender	0.0007^*^	0.0004^*^					
Educational years							
HADA (anxiety)							0.0436^†^
HADD (depression)	0.0341^†^			0.0400^†^		0.0136^†^	

^*∗*^ *P* < 0.001.

^#^ *P* < 0.01.

^†^ *P* < 0.05.

Table 2(a): each line corresponds to the *P* values, corrected *P* values, and adjusted-*R* of each model (*n* = 7) subjected to regression and cross-validation analysis.

Table 2(b): each line corresponds to the *P* values of each predictor for every regression model performed.

The different symbols denote the difference in significance: ^*∗*^highest significance (*P* < 0.001), ^#^middle range significance (*P* < 0.01), ^†^low significance (*P* < 0.05), and ^‡^nonsignificant predictor (*P* > 0.05).
