# Supplementary material for: Multicontrast MRI Quantification of Focal Inflammation and Degeneration in Multiple Sclerosis
Source: Biomed Res Int. 2015 Jul 29;2015:569123. doi: 10.1155/2015/569123 (PMC4532805; doi:10.1155/2015/569123)
Supplement: Supplementary file 1 — Description of the MRI acquisition protocol. TR: repetition time; TE: echo time; FoV: field of view. [file 569123.f1.pdf]

Supplementary data :

Sequence	Protocol	Acquisition time
T2* (MT)	TR/TE = 47/1.23 ms; 32 echoes, ΔTE = 1.23 ms; Voxel size = 1.6×1.6×1.6 mm^3 ; FoV=136×136×112	11' 16''
T2	TR = 5000 ms, 21 echoes with ΔTE = 9 ms; Voxel size = 1.1×1.1×4.0 mm^3, FoV=160×192×30	3'15"
MP2RAGE	TR/TE = 5000/2.89, IT1 = 700 ms, IT2=2500 ms; Voxel size= 1.0×1.0×1.2 mm^3, FA = 4°;FoV = 256×240×176	8' 22''
3DFLAIR	TR/TE/TI = 5000/394/1800 ms; Voxel size = 1.0×1.0×1.2 mm^3; FoV = 256×240×176	6' 27''
DIR	TR/TE/TI = 10000/218/3650 ms; Voxel size= 1.1×1.0×1.2 mm^3; FoV = 240×256×160	12' 52''
MPRAGE	TR/TE = 2300/2.98 ms; Voxel size= 1.0×1.0×1.2 mm^3; FoV= 256×240×160,	5'12"
	Total acquisition time	1h 08'

Table 1s. Supplementary data. MRI acquisition protocol. TR: repetition time; TE: echo time; FoV: field of view.
